# Honey

**Published:** 1870-10

**Authors:** 


### HONEY.

Such a delicious article for the table should be one of the products of every well-conducted farm, and yet very few farmers give it any attention. There ought to be in this country a hundred hives where there is now but one. We are glad to notice a monthly publication, one dollar a year—H. A. King & Co., 240 Broadway, New York—called the *Bee-Keepers' Journal*, with a large amount of valuable information besides about poultry, the farm and garden. It merits, and, we trust, it will inspire, an ambition in our farmers to raise bees and make money in one of the easiest ways possible on the farm.

